# Incidental Malignancies in the Emergency Department: Missed Diagnoses, Radiological Difficulty, and a Risk-Based Framework for Follow-Up

**DOI:** 10.3390/jcm15135071

**Published:** 2026-06-29

**Authors:** Hiromi Takayasu, Kazuyuki Miyamoto, Mako Kitazono, Nobuyuki Takeyama, Hiroki Yamaga, Yuki Kaki, Atsuo Maeda, Ayako Iijima, Jun Sasaki, Kenji Dohi

**Affiliations:** 1 Department of Emergency, Critical Care Medicine and Disaster Medicine, Showa Medical University Fujigaoka Hospital, 1-30 Fujigaoka Aoba-ku, Yokohama City 227-8501, Kanagawa, Japan; thiromi@med.showa-u.ac.jp (H.T.); mako.s_0815@med.showa-u.ac.jp (M.K.); h.yamaga@med.showa-u.ac.jp (H.Y.); yuki.kaki5@med.showa-u.ac.jp (Y.K.); atsuo@med.showa-u.ac.jp (A.M.); kprsto.a@med.showa-u.ac.jp (A.I.); 2 Department of Emergency, Critical Care Medicine and Disaster Medicine, Showa Medical University School of Medicine, 1-5-8 Hatanodai, Shinagawa-ku, Tokyo 142-8666, Japan; jun-sa@da2.so-net.ne.jp (J.S.); kdop@med.showa-u.ac.jp (K.D.); 3 Department of Radiology, Showa Medical University Fujigaoka Hospital, 1-30 Fujigaoka, Aoba-ku, Yokohama City 227-8501, Kanagawa, Japan; 4 Department of Emergency & Critical Care Center, Kawakita General Hospital, 1-6-1 Asagaya-kita, Suginami-ku, Tokyo 166-8588, Japan; 5 Department of Emergency and Disaster Medicine, Showa Medical University Koto Toyosu Hospital, 5-1-38 Toyosu, Koto-ku, Tokyo 135-8577, Japan

**Keywords:** incidental malignancy, emergency department, computed tomography, missed diagnosis, radiological interpretation, follow-up

## Abstract

**Background**: Incidental malignancies on emergency department (ED) CT may go unrecognized, risking delayed diagnosis. We investigated their prevalence, recognition rate, and follow-up risk stratification. **Methods**: We retrospectively reviewed 15,347 CT reports from a tertiary ED (April 2016–March 2019). To determine whether missed diagnoses reflected inherent radiological difficulty or situational factors, ten board-certified emergency physicians completed a standardized 18-case interpretation test under off-duty conditions. **Results**: Incidental malignancies were identified in 25 patients (0.16%); lung carcinoma was most common (28%). Of 25 patients, 16 (64%) had malignancy recognized at the initial ED visit; 9 (36%) were missed. One discharged new patient without scheduled follow-up represented the highest-risk group for loss to follow-up. Diagnostic accuracy ranged from 0/10 to 9/10 (Cochran’s Q test, *p* < 0.001). Cases 1, 4, and 7 were unidentified by all evaluators (*p* = 0.002 vs. Case 16, McNemar test with Bonferroni correction). No significant inter-physician difference was observed (*p* = 0.73, Friedman test). **Conclusions**: Incidental malignancies occurred in 0.16% of ED CT examinations, with 36% missed at initial presentation. Missed diagnoses reflected inherent radiological difficulty and systemic factors rather than individual expertise. Discharged new patients without scheduled follow-up represent the highest-risk group, underscoring the need for systems-based interventions.

## 1. Introduction

The use of computed tomography (CT) in the emergency department (ED) has continued to increase significantly in recent years, with the number of CT scans obtained in ED patients estimated to exceed 30 million annually in the United States alone. This expanding utilization has led to a growing recognition of incidental findings—abnormalities detected on imaging performed for an unrelated reason [1,2]. A recent meta-analysis reported a pooled prevalence of 31.3% for any incidental finding on ED CT studies [1], consistent with a 1-year single-center review reporting a similarly high incidence of incidental findings on emergency abdominal CT [3], and it is estimated that actionable incidental findings occur in 5% to 30% of imaging studies [4]. Among these, incidental malignancies represent a clinically significant subset, offering a potential opportunity for early cancer detection and treatment.

However, the ED presents unique challenges for the management of incidental findings. Emergency physicians who order imaging tests do not have the same ongoing relationship with the patient as a primary care physician and are typically not involved in follow-up care [4]. The high-volume, time-pressured environment of the ED may predispose to cognitive biases such as satisfaction of search, in which attention to incidental findings is diminished once a primary diagnosis has been established. Furthermore, current systems of referral and linkage often do not meet clinical needs, with large portions of ED-identified cancers having marginal or no follow-up [5]. These factors collectively create a risk that incidentally discovered malignancies may go unrecognized or unreported, potentially leading to delayed diagnosis and worse patient outcomes.

Despite the clinical importance of this issue, data on the actual incidence of incidental malignancies in the ED, the rate at which they are recognized by emergency physicians, and the factors that determine whether appropriate follow-up is achieved remain limited. In this study, we retrospectively reviewed 15,347 CT reports from a tertiary ED over a three-year period to determine the prevalence and clinical characteristics of incidentally discovered malignancies. We further investigated the rate of recognition by emergency physicians and the subsequent referral for diagnostic evaluation. Finally, to assess whether missed diagnoses were attributable to inherent radiological difficulty or to situational factors, we conducted a standardized radiological interpretation performance test among ten board-certified emergency physicians using a set of 18 CT cases comprising the nine missed malignancies and nine matched control cases.

## 2. Materials and Methods

### 2.1. Study Design and Ethical Consideration

This retrospective study was conducted using computed tomography (CT) interpretation reports obtained from the Emergency Department (ED). The study protocol was reviewed and approved by the Clinical Trial Review Board of Showa Medical University (Approval No. #F2019C84). The study was performed in accordance with the ethical principles of the Declaration of Helsinki and adhered to the Council for International Organizations of Medical Sciences (CIOMS) International Ethical Guidelines for Health-related Research Involving Humans.

### 2.2. Study Setting

This study was conducted at the Emergency Department (ED) of a university hospital, a certified tertiary critical care center. The medical services were provided by a team of five certified emergency physicians and internists and surgeons from within the hospital, who work in shifts. The department operated on a two-shift system: a day shift (08:30–17:00) and a night shift (17:00–08:30). During the day shift, the medical team primarily managed patients transported via ambulance; during the night shift, they treated both ambulance-transported and walk-in patients. Each shift was operated by three to four attending physicians and two residents.

### 2.3. Radiological Interpretation and Reporting System

The ED was equipped with a dedicated computed tomography (CT) scanner (LightSpeed VCT, GE HealthCare, Chicago, IL, USA), facilitating immediate imaging 24 h a day. To ensure diagnostic accuracy, all CT images underwent a mandatory double-check interpretation by two board-certified radiologists during the subsequent day shift, achieving a 100% formal interpretation rate. The radiologists reviewed the clinical information in the imaging request before interpreting each scan. When a potential incidental malignancy was identified, the radiologists reviewed the medical record to determine whether the ED physician had already recognized the finding; if not, they directly contacted the attending ED physician to communicate the finding. The turnaround time for definitive reports was typically the next business day on weekdays, though it could extend to three days during weekends or public holidays. The attending physicians on duty were responsible for reviewing these formal reports and contacting patients if further clinical intervention, such as for incidental malignancies, was deemed necessary.

### 2.4. Patients Selection and Data Collection

**Figure 1:**
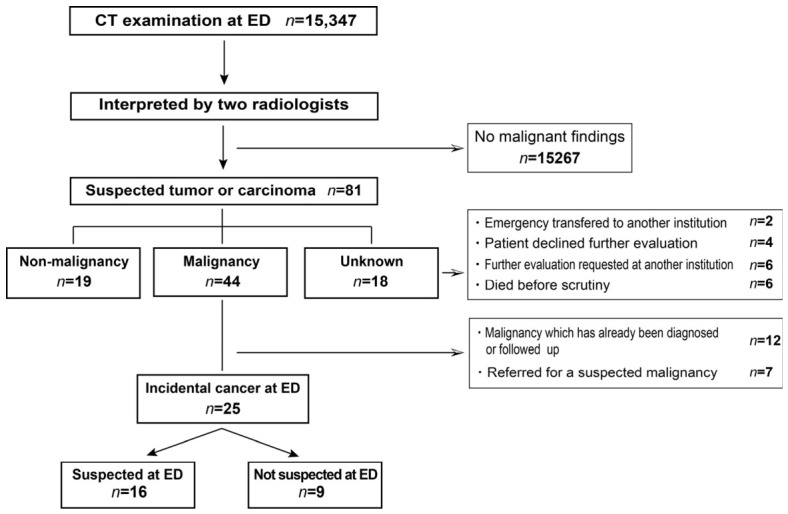
Patient selection flowchart. Of 15,347 CT examinations performed in the emergency department between April 2016 and March 2019, radiological reports flagging potential malignancy were identified in 81 cases by two administrative staff members. Subsequent medical record review excluded 56 cases (19 benign lesions and 37 previously diagnosed malignancies), yielding 25 patients with newly identified incidental malignancies. ED, emergency department; CT, computed tomography.

Between April 2016 and March 2019, 15,347 computed tomography (CT) reports from the Emergency Department (ED) were retrospectively reviewed (Figure 1).

To identify incidental malignancies, two administrative staff members independently screened all reports, initially identifying 81 patients with suspected tumors. Subsequent medical record reviews revealed that 19 of these cases were benign.

Of the 81 patients with suspected malignancy, 18 were classified as having an unknown outcome and were excluded from further analysis due to: (1) emergency transfer to another institution (*n* = 2); (2) refusal of further evaluation by the patient or family (*n* = 4); (3) preference for follow-up at another institution (*n* = 6); and (4) death before scrutiny could be completed (*n* = 6). These 18 patients were distinct from the 25 patients with confirmed incidental malignancy.

Of the remaining 44 patients, 19 were excluded as they had a prior diagnosis of malignancy or were referred to the ED for suspected tumors. Ultimately, 25 patients were identified as having incidental cancers discovered in the ED.

### 2.5. Outcome Measures and Clinical Evaluation

The primary outcome of this study was to determine the prevalence and clinical characteristics of incidentally discovered malignancies identified on computed tomography (CT) scans performed in the emergency department (ED). In addition, we assessed whether attending emergency physicians recognized the potential for malignancy at the time of the initial ED visit and initiated appropriate diagnostic follow-up, based on a comprehensive review of medical records. We also identified cases in which the potential for malignancy was overlooked or not documented by emergency physicians and evaluated patients who were ultimately diagnosed with malignancy following formal radiologist interpretation. For the purposes of this study, a malignancy was considered “recognized at the initial ED visit” if the attending emergency physician documented the finding in the medical record, communicated it to the patient, or initiated a referral for further evaluation based on the CT findings. This determination was made through a comprehensive review of medical records, including ED physician notes, discharge summaries, and referral documents, conducted independently by two investigators (H.T. and K.M.). Discrepancies were resolved by consensus.

To further stratify the risk of inadequate follow-up, patients were classified according to two clinical factors: (1) disposition at the ED visit (admitted vs. discharged) and (2) patient status (established vs. new patient). Patients who were discharged home were considered at higher risk for loss to follow-up compared to those who were admitted, as radiological findings could be communicated directly during hospitalization. Among discharged patients, those who were new patients without scheduled follow-up appointments were considered to be at the highest risk for missed follow-up, as contact relied solely on telephone outreach, which may be unreliable due to incorrect contact information or impaired comprehension, particularly in elderly patients.

### 2.6. Radiological Interpretation Performance Test

**Figure 2:**
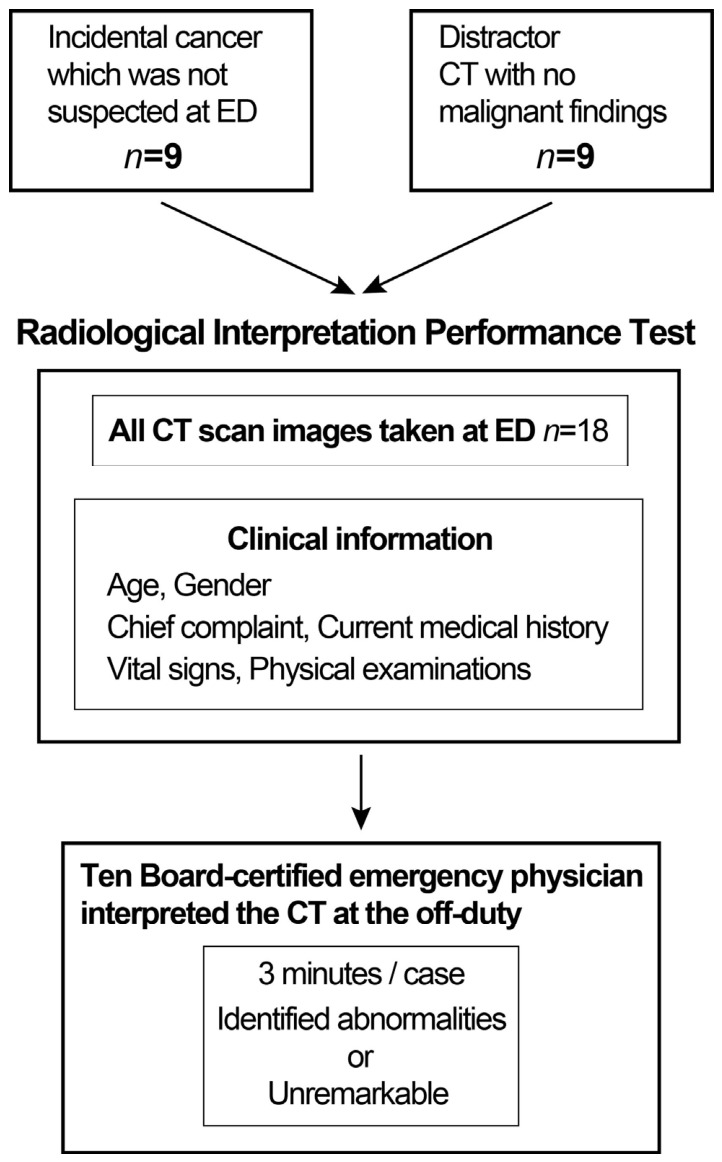
Study protocol for the radiological interpretation performance test. Nine cases of incidental malignancy missed at the initial emergency department visit were paired with nine sex-matched control cases with comparable ages (Distractor group), yielding 18 test cases. Ten board-certified emergency physicians independently reviewed each case under off-duty conditions, with three minutes allocated per case. Clinical information including age, sex, chief complaint, past medical history, and the purpose of imaging was provided for each case.

To determine whether missed diagnoses in the emergency department (ED) were attributable to the inherent diagnostic difficulty of radiological findings or to clinical oversight, we conducted a validation study using a standardized radiological interpretation test. Nine cases of incidentally discovered malignancies that had been missed in the ED were selected and paired with nine sex-matched control cases with comparable ages (Distractor group: 69.4 ± 8.8 years vs. Cancer group: 74.9 ± 6.2 years, *p* = 0.288) (e.g., pneumonia or cholecystitis without malignancy), yielding a total of 18 test cases (the study flow is shown in Figure 2). The distractor cases consisted of non-malignant diagnoses (e.g., pneumonia, cholecystitis) and were included specifically to prevent evaluators from recognizing that all cases contained malignancies, thereby reducing detection bias. Nine cases of incidentally discovered malignancies that had been missed in the ED were selected and paired with nine sex-matched control cases with comparable ages (Distractor group: 69.4 ± 8.8 years vs. Cancer group: 74.9 ± 6.2 years, *p* = 0.288) (e.g., pneumonia or cholecystitis without malignancy), yielding a total of 18 test cases (the study flow is shown in Figure 2).

The clinical and radiological characteristics of all 18 test cases are summarized in Table 1.

Table board-certified emergency physicians participated in the test under calm, non–working-hour conditions. For each case, relevant clinical information—including age, sex, chief complaint, past medical history, history of present illness, physical examination findings, and the purpose of imaging—was provided. Each physician was allotted three minutes per case for image interpretation. We evaluated whether the physicians were able to identify the potential presence of malignancy under these conditions. In addition, we analyzed imaging characteristics that were statistically associated with diagnostic difficulty. Furthermore, we assessed inter-observer variability in diagnostic performance among the participating physicians.

### 2.7. Statistical Analysis

Statistical analyses were performed using JMP^®^ Pro 16 (SAS Institute Inc., Cary, NC, USA). Categorical variables were expressed as frequencies and percentages. To compare the identification rates (correct vs. incorrect) among the nine test cases, Cochran’s Q test was employed, as the data consisted of related dichotomous variables obtained from the same group of observers. For post hoc pairwise comparisons, the McNemar test with Bonferroni correction was applied to compare the three cases with the lowest diagnostic accuracy (Cases 1, 4, and 7) against the case with the highest diagnostic accuracy (Case 16), resulting in three pairwise comparisons and an adjusted significance level of α = 0.017.

To evaluate whether diagnostic performance varied significantly among the ten physicians, the Friedman test was used to compare the overall identification scores. All statistical tests were two-tailed, and a *p*-value of < 0.05 was considered statistically significant, except for the post hoc analyses, where the adjusted significance level was applied. Kendall’s W was calculated as a measure of inter-rater agreement and effect size for the Friedman test.

## 3. Results

### 3.1. Incidental Malignancies at the Emergency Department

Incidentally detected malignancies in the emergency department were identified in 25 patients (Table 2), corresponding to an incidence rate of 0.16% (25/15,347).

The most common malignancy was lung carcinoma, identified in 7 patients (28%), followed by bladder carcinoma in 3 patients (12%), pancreatic carcinoma in 3 patients (12%), gastric carcinoma in 2 patients (8%), colorectal carcinoma in 2 patients (8%), and renal cell carcinoma in 2 patients (8%). The remaining cases included breast carcinoma, malignant lymphoma, ovarian carcinoma, prostatic adenocarcinoma, cholangiocarcinoma, and thyroid cancer with thoracic vertebral metastasis (1 patient each). The full distribution of cancer types is presented in Table 2.

Among the 25 patients (Table 3), 17 were male and 8 were female, with a median age of 77 years (interquartile range [IQR], 70–82 years).

In 16 patients, malignancy was suspected at the time of the emergency department visit, and diagnostic evaluation was initiated concurrently. In contrast, in the remaining 9 patients (Table 4), the possibility of malignancy was not recognized during the initial emergency department encounter, and further diagnostic evaluation was subsequently performed based on radiology reports issued after the visit. Of the 25 patients, 20 patients were admitted on the same day, whereas 5 patients were discharged home. Additionally, 18 patients were new patients, and 7 patients were established patients at our institution. Of the 5 discharged patients, 4 were established patients with scheduled follow-up appointments. Only 1 patient (Case 1; 81-year-old female, gastric carcinoma) was a new patient without a scheduled follow-up appointment. Although this patient represents the highest-risk profile for loss to follow-up based on clinical reasoning, drawing definitive conclusions from a single case is inherently limited, and this observation should be interpreted as a hypothesis requiring validation in larger multicenter studies.

### 3.2. Identification Rates and Diagnostic Difficulty

**Figure 3:**
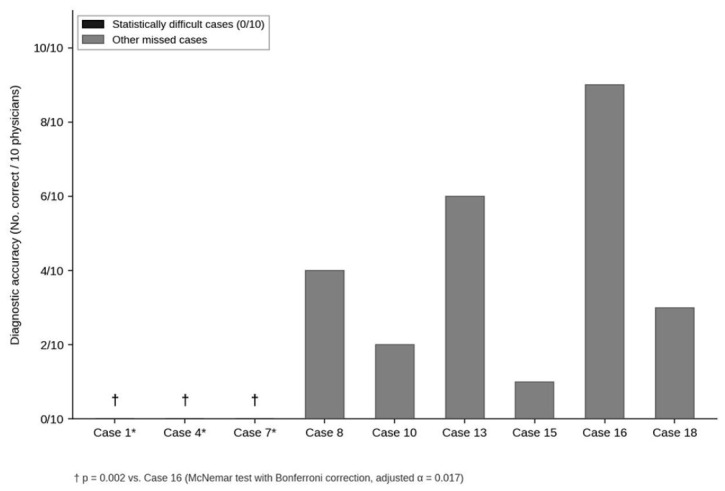
Diagnostic accuracy of ten emergency physicians for each of the nine missed malignancy cases. The proportion of physicians correctly identifying each case is shown. Cases 1, 4, and 7 had a diagnostic accuracy of 0/10. Case 16 achieved the highest accuracy (9/10). A statistically significant difference in diagnostic accuracy was observed across cases (Cochran’s Q test, *p* < 0.001). Post hoc analysis confirmed that Cases 1, 4, and 7 were significantly more difficult than Case 16 (McNemar test with Bonferroni correction, adjusted α = 0.017; *p* = 0.002 for all three comparisons). The asterisk (*) denotes statistical significance (*p* < 0.017 after Bonferroni correction).

The image interpretation results of nine cases with incidental malignancy were analyzed based on assessments by ten emergency physicians. A statistically significant difference in diagnostic accuracy was observed among the cases (Cochran’s Q test, *p* < 0.001) (Figure 3).

In particular, Cases 1, 4, and 7 were not identified as having malignant disease by any of the evaluators and were therefore considered statistically difficult to diagnose (Table 5). The remaining cases showed diagnostic accuracies of 4/10 (Case 8, *p* = 0.031), 2/10 (Case 10, *p* = 0.008), 6/10 (Case 13, *p* = 0.125), 1/10 (Case 15, *p* = 0.004), and 3/10 (Case 18, *p* = 0.016) compared to Case 16, though none reached the Bonferroni-corrected significance threshold of α = 0.017.

In contrast, malignant disease was correctly identified by all evaluators for Case 16. Post hoc analysis (McNemar test with Bonferroni correction, adjusted α = 0.017) confirmed that Cases 1, 4, and 7 were significantly more difficult to diagnose compared to Case 16 (b = 10, c = 0, *p* = 0.002 for all three comparisons), where b represents the number of physicians who correctly identified Case 16 but not the target case, and c represents the reverse.

### 3.3. Physician Performance Consistency

There was no significant difference in diagnostic performance among the ten physicians (*p* = 0.73, Friedman test) (Table 6). Kendall’s W was 0.027, indicating very weak inter-rater agreement, which suggests that diagnostic performance was primarily driven by case-specific radiological characteristics rather than individual physician expertise.

The number of correctly identified cases per physician ranged from 2 to 4 (out of 9), with a median score [Q1 Q3] of 2 [2, 3]. This indicates that the missed diagnoses were likely attributable to the radiological characteristics of the specific cases or systemic factors rather than individual differences in physician expertise.

### 3.4. Images in Which Malignancy Was Not Identified in the Emergency Department

**Figure 4:**
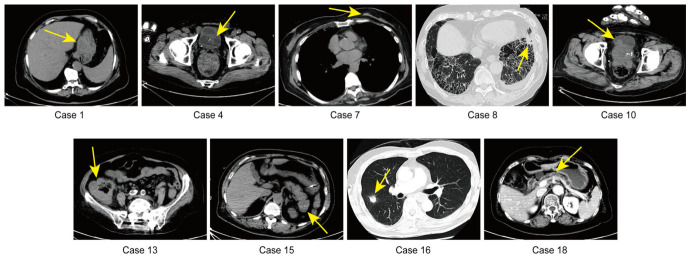
Computed tomography images of the nine incidental malignancies missed in the emergency department. (Case 1) An 81-year-old female presenting with chest discomfort and nausea. CT demonstrates subtle thickening of the upper gastric wall with mild adjacent lymph node enlargement (arrow), subsequently confirmed as gastric carcinoma. (Case 4) A 79-year-old male presenting with back pain and posterior neck pain following a traffic accident. A whole-body CT incidentally demonstrates a protruding lesion with calcification within the urinary bladder (arrow), subsequently confirmed as bladder carcinoma. (Case 7) A 69-year-old female presenting with hemoptysis. CT demonstrates a nodule in the left mammary gland (arrow), subsequently confirmed as breast carcinoma. (Case 8) A 75-year-old male presenting with cough and dyspnea. CT demonstrates an enlarging peripheral nodule in the left pulmonary lingula (arrow), subsequently confirmed as lung carcinoma. (Case 10) An 82-year-old male presenting with fever and fatigue. CT demonstrates a 17 mm elevated lesion at the right bladder neck (arrow), subsequently confirmed as bladder carcinoma. (Case 13) An 82-year-old female presenting with hematochezia. CT demonstrates irregular wall thickening of the cecum (arrow), subsequently confirmed as malignant lymphoma of the ileocecal region. (Case 15) A 69-year-old male presenting with dyspnea. CT demonstrates a solid mass in the pancreatic tail (arrow), subsequently confirmed as pancreatic carcinoma. (Case 16) A 68-year-old male presenting with hematemesis and melena. CT demonstrates a solid nodule in the right upper lobe (arrow), subsequently confirmed as lung carcinoma. (Case 18) A 69-year-old female presenting with right upper quadrant pain. CT demonstrates main pancreatic duct dilation with pancreatic atrophy and multiple cysts (arrow), subsequently confirmed as cholangiocarcinoma.

The CT images of the nine missed cases are presented in Figure 4, and their clinical characteristics are summarized in Table 4. Clinical follow-up was available for all nine missed cases. Six patients were admitted and had their radiology reports reviewed during hospitalization; three were discharged home and subsequently contacted for follow-up. All nine patients underwent further evaluation after the incidental malignancy was identified through formal radiology reporting. Among the nine patients, Case 4 (bladder carcinoma) died during the index hospitalization due to urinary tract infection, unrelated to the malignancy. Case 13 (malignant lymphoma) responded well to chemotherapy but died approximately two years later from cardiopulmonary arrest of unknown cause. Case 18 (cholangiocarcinoma) was referred to another institution for surgery, and detailed outcomes were unavailable. The remaining six patients were alive at the time of data collection.

Cases 1, 4, and 7 had a diagnostic accuracy of 0/10, meaning that none of the ten evaluators identified the malignancy under off-duty conditions. In contrast, Case 16 achieved the highest diagnostic accuracy (9/10) among the nine missed cases.

## 4. Discussion

In this retrospective study of 15,347 CT reports obtained from a tertiary emergency department over a three-year period, incidental malignancies were identified in 25 patients (0.16%). Of these, 16 patients (64%) had their malignancy recognized at the time of the ED visit and were promptly referred for further evaluation. However, in the remaining 9 patients (36%), the potential for malignancy was not recognized during the initial ED encounter and was subsequently identified through formal radiological reports. A standardized radiological interpretation test revealed that three of the nine missed cases (Cases 1, 4, and 7) were not identified by any of ten board-certified emergency physicians under off-duty conditions, suggesting inherent radiological difficulty. No significant difference in diagnostic performance was observed among the ten physicians, indicating that missed diagnoses were attributable to case-specific radiological characteristics or systemic factors rather than individual physician expertise. Furthermore, risk stratification identified that discharged new patients without scheduled follow-up represent the most vulnerable group for loss to follow-up.

The incidence of incidental malignancy in this study was 0.16% (25/15,347 CT examinations). While direct comparisons are difficult due to differences in study populations and definitions, this rate is consistent with the broader literature on incidental findings in the ED. Of the 81 patients flagged as having suspected malignancy, 19 were subsequently found to have benign lesions and 25 were confirmed as malignant, yielding a positive predictive value (PPV) of 30.9% (25/81). This figure is clinically relevant, as it reflects the proportion of radiologically suspected malignancies that ultimately represent true incidental cancers in an ED setting. A recent meta-analysis reported a pooled prevalence of 31.3% for any incidental finding on ED CT studies, and incidental finding rates of 34% to 43% have been reported in abdominal CT scans of trauma patients [6,7]. However, the majority of these findings are benign; confirmed malignancies represent only a small subset. The relatively low incidence of confirmed malignancy in our study (0.16%) reflects this reality, as the majority of suspected tumors identified on screening were subsequently found to be benign (19/81 cases). Notably, lung carcinoma was the most frequently identified malignancy (28%), consistent with the known sensitivity of CT for pulmonary nodule detection. The relatively high proportion of elderly patients (median age 77 years) in this cohort likely reflects the demographic characteristics of our ED population and the well-established association between advanced age and cancer incidence.

In this study, 9 of 25 patients (36%) with incidental malignancy were not recognized at the time of the initial ED visit, with the diagnosis subsequently established through formal radiological reports. A scoping review has highlighted that many cancers are identified during the ED workup for unrelated complaints, most often as incidental findings on CT scans, and that large portions of ED-identified cancers have marginal or no follow-up [5]; however, specific follow-up rates were not quantified in that review. Our findings are consistent with this observation.

An important factor influencing detection rates was the relationship between the chief complaint and the location of the malignancy. Among the 25 patients with incidental malignancy, only one patient (Case 14) presented via a trauma mechanism (traffic accident). Given this small number, a formal subgroup analysis of trauma versus non-trauma presentations was not feasible. However, the susceptibility to missed diagnoses in trauma settings—where clinician attention is naturally directed toward acute injuries—is well recognized in the literature [8] and warrants further investigation in larger, dedicated trauma cohorts. In cases where the chief complaint was anatomically related to the site of malignancy—such as hematuria leading to the discovery of bladder carcinoma, or hematochezia prompting detection of colorectal lesions—the malignancy was more likely to be recognized during the ED visit. In contrast, incidental malignancies located in organs unrelated to the presenting complaint were more susceptible to being overlooked.

The results of the radiological interpretation performance test provide further insight into the nature of these missed diagnoses. Cases 1, 4, and 7—in which none of the ten evaluators identified the malignancy—suggest inherent radiological difficulty. In contrast, Case 16, which was correctly identified by 9 of 10 evaluators under off-duty conditions, raises the possibility that its missed diagnosis in the ED was attributable to situational factors. In a study classifying types of radiologic diagnostic errors, 22% were related to satisfaction of search, making it the most common cognitive bias in diagnostic radiology [9]. In Case 4, where a bladder carcinoma was missed during a whole-body trauma CT in the context of multiple acute cervical fractures, this phenomenon of satisfaction of search—defined as decreased vigilance and awareness of additional abnormalities after the first abnormality has been identified [9]—may have contributed to the missed diagnosis. Such cognitive biases are particularly relevant in the ED setting, where high case volume, high acuity, and physician fatigue intersect [10,11]. Notably, in Case 4, the formal radiology report did identify the bladder lesion; it was the ED physician who did not recognize it during the initial assessment, while attention was directed toward the acute traumatic injuries. This further supports the interpretation that the missed diagnosis was attributable to situational factors rather than inherent radiological difficulty.

Notably, no significant difference in diagnostic performance was observed among the ten participating physicians (*p* = 0.73, Friedman test), indicating that missed diagnoses were not attributable to individual physician expertise. This finding suggests that the underlying causes are systemic—related either to the inherent radiological characteristics of the cases or to the environmental conditions of the ED—rather than to individual competence.

Our risk stratification framework identified three clinically meaningful groups based on disposition and patient status. Admitted patients, regardless of whether their malignancy was recognized at the time of the ED visit, were considered at low risk for loss to follow-up, as radiological findings could be communicated during hospitalization. Among the 5 discharged patients, 4 were established patients with scheduled follow-up appointments, providing a natural opportunity to communicate and act on radiological findings. However, 1 patient—a new patient discharged without a scheduled follow-up appointment (Case 1; 81-year-old female, gastric carcinoma, diagnostic accuracy 0/10)—represented the highest-risk category, as follow-up relied entirely on telephone outreach.

Many incidental findings in emergency department patients may not receive the follow-up that they should [4], because the patient may have come in for a different complaint, and care may occur at any time of the day or week, making it difficult to ensure that incidental findings are reliably communicated and followed. This challenge is compounded in elderly patients, in whom telephone-based communication may be particularly unreliable due to hearing impairment, cognitive decline, or incorrect contact information [12,13].

There was strong consensus in the literature that during the initial ED encounter any incidental finding should be verbally conveyed to the patient [4] by the clinician before discharge, and that written communication about the finding should be included in any discharge paperwork. Ideally, the patient’s primary care physician should be made aware of any actionable incidental finding and should help coordinate follow-up care [14]. Importantly, the communication of incidental findings is ultimately a systems responsibility as opposed to the responsibility of individual clinicians [15,16]. In our institution, telephone outreach served as the primary mechanism for contacting discharged patients [17,18] with incidental malignancies not recognized at the initial ED visit. While this approach was effective in most cases, it remains vulnerable in elderly, cognitively impaired, or socially disadvantaged or isolated patients. Implementation of systematic tracking mechanisms [19]—such as electronic health record alerts, dedicated nurse navigators, or structured referral pathways—may help mitigate this risk [16,20].

To determine whether missed diagnoses were attributable to inherent radiological difficulty or to situational factors in the ED, we conducted a standardized radiological interpretation performance test under off-duty, non-clinical conditions. The overall diagnostic accuracy ranged from 0/10 to 9/10 across the nine missed cases, with a statistically significant difference among cases (Cochran’s Q test, *p* < 0.001). This wide range indicates that the nine missed cases were not uniformly difficult, but rather represented a spectrum of radiological complexity.

Cases 1, 4, and 7—in which none of the ten evaluators identified the malignancy even under optimal, off-duty conditions—can be considered inherently difficult to diagnose radiologically. In contrast, Case 16, correctly identified by 9 of 10 evaluators, which suggests that its missed diagnosis in the actual ED setting was more likely attributable to situational factors. The original miss of Case 16—a pulmonary nodule on a chest CT performed for upper gastrointestinal bleeding—may reflect such situational factors, as the physician’s attention was directed toward the gastrointestinal findings.

Importantly, no significant difference in diagnostic performance was observed among the ten participating physicians (*p* = 0.73, Friedman test), with individual scores ranging from 2 to 4 correct identifications out of 9 cases (median 2 [IQR 2–3]). The absence of inter-physician variability suggests that the missed diagnoses were not due to individual differences in expertise [21,22], but rather reflect either the inherent difficulty of the radiological findings or systemic environmental factors common to all physicians working in the ED. These findings underscore that addressing missed diagnoses of incidental malignancy in the ED requires systemic interventions rather than individual physician training alone.

This study has several limitations. First, it was conducted at a single tertiary care emergency department in Japan, which may limit the generalizability of our findings. The patient demographics, CT utilization patterns, and radiological reporting systems may differ across institutions and countries. Second, although the overall CT dataset was large (15,347 examinations), the final analytic sample consisted of only 25 confirmed incidental malignancies, with 9 classified as missed. This small sample size severely limits the statistical power of the study and makes generalization to broader clinical settings difficult. In particular, the highest-risk group identified in our risk stratification framework—new patients discharged without scheduled follow-up—comprised only one patient (Case 1). While this observation is clinically plausible, drawing definitive conclusions from a single case is inherently limited, and this finding should be regarded as a hypothesis-generating observation requiring validation in larger, multicenter cohorts. Third, the retrospective design introduces the potential for selection bias and incomplete data. The classification of whether a malignancy was recognized at the initial ED visit was based on chart review; subtle or undocumented clinical judgments may not have been fully captured. Fourth, the radiological interpretation performance test was conducted under off-duty, non-clinical conditions, which differ fundamentally from the high-stress environment of actual ED practice. Furthermore, because participants were aware they were taking part in an interpretation test, they likely exhibited heightened vigilance compared to routine clinical practice, potentially overestimating real-world diagnostic capability. Fifth, the test cases were selected exclusively from the 9 missed malignancy cases, which by definition represent the most diagnostically challenging subset. This selection bias may have inflated the apparent radiological difficulty of missed diagnoses. Sixth, among the 25 patients with incidental malignancy, only one presented via a trauma mechanism, precluding a formal subgroup analysis of trauma versus non-trauma presentations. Seventh, the study period (April 2016–March 2019) predates the widespread adoption of artificial intelligence-assisted image interpretation tools, which may have implications for the current applicability of these findings.

In conclusion, this study demonstrates that incidental malignancies are identified in a small but clinically significant proportion of ED CT examinations [23], and that a substantial number of these are not recognized at the time of the initial ED visit. The radiological interpretation performance test revealed that missed diagnoses are attributable to a combination of inherent radiological difficulty and situational factors in the ED environment, rather than to individual physician expertise. These findings underscore the importance of a systems-based approach to managing incidental malignancies in the ED. Risk stratification based on patient disposition and follow-up status provides a practical framework for identifying patients most vulnerable to loss to follow-up. In particular, new patients who are discharged without scheduled follow-up appointments represent the highest-risk group and warrant targeted interventions, such as structured telephone outreach protocols, electronic health record alert systems, or dedicated nurse navigator programs. The double-check radiological reporting system employed at our institution proved effective in identifying incidental malignancies that were initially missed by ED physicians, and represents a potentially transferable model for other institutions.

## 5. Conclusions

Incidental malignancies were identified in 0.16% of CT examinations performed in the emergency department, with 36% of cases not recognized at the time of the initial ED visit. A standardized radiological interpretation performance test demonstrated that missed diagnoses were attributable to a combination of inherent radiological difficulty and situational factors, rather than individual physician expertise, as no significant difference in diagnostic performance was observed among the ten participating physicians. Risk stratification identified discharged new patients without scheduled follow-up as the highest-risk group for loss to follow-up. These findings highlight the importance of a systems-based approach—including mandatory secondary radiological interpretation, structured follow-up protocols, and targeted outreach for high-risk patients—to ensure that incidentally discovered malignancies in the ED are appropriately communicated and followed up with.

## Figures and Tables

**Table 1:** Clinical and Radiological Characteristics of the 18 Test Cases Used in the Radiological Interpretation Performance Test.

Case	Age	Sex	Chief Complaint	CT Range	Radiological Findings (Summary)	Final Diagnosis
1 *	81	F	Chest discomfort, Nausea	Chest–Pelvis	Thickening of the upper gastric wall with mild adjacent lymph node enlargement; gastric carcinoma cannot be excluded	Gastric cancer
2	76	M	Abdominal pain	Abdomen–Pelvis	Unremarkable	Within normal limits
3	77	M	Fever, Shaking chills	Abdomen–Pelvis	Hydronephrosis, prostatic enlargement, bilateral pleural effusion	Pyelonephritis
4 *	79	M	Back pain, Neck pain (Traffic accident)	Whole-body	Cervical and thoracic vertebral fractures; protruding bladder lesion with calcification suspicious for bladder cancer	Bladder cancer
5	70	F	Fever, Dyspnea	Chest–Pelvis	Poor aeration of the right lower lobe with bilateral infiltrates	Aspiration pneumonia
6	61	F	Right shoulder pain, Back pain	Chest	Unremarkable	Within normal limits
7 *	69	F	Hemoptysis	Chest	Granular opacities and bronchiectasis consistent with NTM infection; nodule in the left mammary gland suspicious for breast cancer	Breast cancer
8 *	75	M	Cough, Dyspnea	Chest	Stable idiopathic pulmonary fibrosis; enlarging peripheral nodule in the left lingula suspicious for malignancy	Lung cancer
9	72	M	Fever, Dyspnea	Chest–Pelvis	Pulmonary emphysema with suspected right-sided pneumonia; hepatic steatosis; Th12 compression fracture	Pulmonary emphysema
10 *	82	M	Fever, Fatigue	Whole-body	17 mm elevated lesion at the bladder neck highly suspicious for bladder cancer; left renal stone, gallstones, benign prostatic hyperplasia	Bladder cancer
11	73	M	Abdominal pain, Vomiting	Abdomen–Pelvis	Unremarkable	Within normal limits
12	80	F	Fever, Shaking chills	Chest–Pelvis	Choledocholithiasis with suspected cholangitis; mild main pancreatic duct dilation; thoracoabdominal aortic aneurysm	Cholangitis
13 *	82	F	Hematochezia	Chest–Pelvis	Irregular wall thickening of the cecum	Malignant lymphoma
14	63	F	Neck pain (Traffic accident)	Whole-body	Unremarkable	Within normal limits
15 *	69	M	Dyspnea	Whole-body	Bilateral pleural effusions and ground-glass opacities; solid mass in the pancreatic tail suspicious for malignancy	Pancreatic cancer
16 *	68	M	Hematemesis and melena	Chest–Pelvis	Hyperattenuating area in the stomach; solid nodule in the right upper lobe suspicious for lung cancer	Lung cancer
17	53	M	Vomiting, Diarrhea, Headache	Whole-body	Unremarkable	Within normal limits
18 *	69	F	Right upper quadrant pain	Chest–Pelvis	Main pancreatic duct dilation, pancreatic atrophy, and multiple cysts suspicious for mixed-type IPMN; thyromegaly; cholelithiasis	Cholangiocarcinoma

* Incidental cancer cases. NTM, non-tuberculous mycobacteria; IPMN, intraductal papillary mucinous neoplasm.

**Table 2:** Profiles of Incidental Malignancies Identified in the Emergency Department (*n* = 25).

Cancer Type	Number of Cases	Percentage (%)
Lung carcinoma	7	28
Bladder carcinoma	3	12
Pancreatic carcinoma	3	12
Gastric carcinoma	2	8
Colorectal carcinoma	2	8
Renal cell carcinoma	2	8
Thyroid cancer with thoracic vertebral metastasis	1	4
Cholangiocarcinoma	1	4
Breast carcinoma	1	4
Prostatic adenocarcinoma	1	4
Malignant lymphoma	1	4
Ovarian carcinoma	1	4
Total	25	100

**Table 3:** Clinical Characteristics of 25 Patients with Incidentally Discovered Malignancies.

Case	Age	Sex	Chief Complaint	Diagnosis at ED	Incidental Cancer	Admission/Discharge	Regular/New	Diagnosed at ED
1	81	F	Chest discomfort, Nausea	Within normal limits	Gastric carcinoma	Discharge	New	No
2	79	M	Back pain, Posterior neck pain (Traffic accident)	Cervical spine fracture	Bladder carcinoma	Admission	New	No
3	69	F	Hemoptysis	Pulmonary Mycobacterium avium complex disease	Breast carcinoma	Discharge	Regular	No
4	75	M	Cough, Dyspnea	Idiopathic pulmonary fibrosis	Lung carcinoma	Discharge	Regular	No
5	82	M	Fever, Fatigue	Urinary tract infection	Bladder carcinoma	Admission	Regular	No
6	82	F	Hematochezia	Ischemic colitis	Malignant lymphoma of the ileocecal region	Admission	New	No
7	69	M	Dyspnea	Congestive heart failure	Pancreatic carcinoma	Admission	New	No
8	68	M	Hematemesis and melena	Hemorrhagic gastric ulcer	Lung carcinoma	Admission	New	No
9	69	F	Right upper quadrant pain	Cholangitis	Cholangiocarcinoma	Admission	New	Yes
10	73	M	Right flank pain	Acute cholangitis, Suspected pancreatic cancer	Pancreatic carcinoma	Admission	New	Yes
11	77	M	Hematochezia	Colonic diverticular bleeding, Bladder tumor suspected	Bladder carcinoma	Admission	Regular	Yes
12	83	M	Right upper quadrant pain	Suspected right lower lobe lung cancer	Lung carcinoma	Admission	New	Yes
13	85	F	Abdominal pain, Hematochezia	Ovarian tumor	Ovarian carcinoma	Admission	New	Yes
14	72	M	Lightheadedness, Anemia	Suspected ascending colon cancer	Colorectal carcinoma	Admission	New	Yes
15	86	M	Hematuria	Suspected renal cancer	Renal cell carcinoma	Admission	New	Yes
16	70	M	Hematuria	Suspected prostate cancer, Postrenal renal failure	Prostatic adenocarcinoma	Admission	New	Yes
17	83	M	Chest pain	Suspected right lung cancer, Pulmonary emphysema	Lung carcinoma	Discharge	Regular	Yes
18	86	F	Inspiratory chest pain	Suspected lung cancer, Pleuritis	Lung carcinoma	Discharge	Regular	Yes
19	75	M	Back pain	Lumbar disc herniation	Renal cell carcinoma	Admission	New	Yes
20	77	M	Abdominal pain	Sigmoid colon cancer suspected	Colorectal carcinoma	Admission	New	Yes
21	80	F	Abdominal pain	Acute cholecystitis	Pancreatic carcinoma	Admission	New	Yes
22	68	F	Abdominal pain, Fever	Acute appendicitis	Gastric carcinoma	Admission	New	Yes
23	82	M	Chest pain	Aortic dissection	Lung carcinoma	Admission	New	Yes
24	64	F	Abdominal pain	Intestinal obstruction	Thyroid cancer with thoracic vertebral metastasis	Admission	New	Yes
25	70	M	Back pain	Lumbar compression fracture	Lung carcinoma	Admission	New	Yes

ED, emergency department.

**Table 4:** Clinical Characteristics of the Nine Incidental Malignancies Missed in the Emergency Department.

Case	Age	Sex	Chief Complaint	Diagnosis at ED	Incidental Cancer	Admission/Discharge	Regular/New
1	81	F	Chest discomfort, Nausea	Within normal limits	Gastric carcinoma	Discharge	New
4	79	M	Back pain, Posterior neck pain (Traffic accident)	Cervical spine fracture	Bladder carcinoma	Admission	New
7	69	F	Hemoptysis	Pulmonary Mycobacterium avium complex disease	Breast carcinoma	Discharge	Regular
8	75	M	Cough, Dyspnea	Idiopathic pulmonary fibrosis	Lung carcinoma	Discharge	Regular
10	82	M	Fever, Fatigue	Urinary tract infection	Bladder carcinoma	Admission	Regular
13	82	F	Hematochezia	Ischemic colitis	Malignant lymphoma of the ileocecal region	Admission	New
15	69	M	Dyspnea	Congestive heart failure	Pancreatic carcinoma	Admission	New
16	68	M	Hematemesis and melena	Hemorrhagic gastric ulcer	Lung carcinoma	Admission	New
18	69	F	Right upper quadrant pain	Cholangitis	Cholangiocarcinoma	Admission	New

Case numbers correspond to those in Table 3. ED, emergency department.

**Table 5:** Diagnostic Accuracy of Ten Emergency Physicians for Each of the Nine Missed Malignancy Cases.

Case	Age	Sex	Chief Complaint	Incidental Cancer	Diagnostic Accuracy	*p* Value *
1	81	F	Chest discomfort, Nausea	Gastric carcinoma	0/10	0.002
4	79	M	Back pain, Posterior neck pain (Traffic accident)	Bladder carcinoma	0/10	0.002
7	69	F	Hemoptysis	Breast carcinoma	0/10	0.002
8	75	M	Cough, Dyspnea	Lung carcinoma	4/10	0.031
10	82	M	Fever, Fatigue	Bladder carcinoma	2/10	0.008
13	82	F	Hematochezia	Malignant lymphoma of the ileocecal region	6/10	0.125
15	69	M	Dyspnea	Pancreatic carcinoma	1/10	0.004
16	68	M	Hematemesis and melena	Lung carcinoma	9/10	—
18	69	F	Right upper quadrant pain	Cholangiocarcinoma	3/10	0.016

* McNemar test with Bonferroni correction (adjusted α = 0.017) vs. Case 16 (reference). —, reference case.

**Table 6:** Individual Diagnostic Performance of Ten Emergency Physicians Across Nine Missed Malignancy Cases.

Physician	Case 1	Case 4	Case 7	Case 8	Case 10	Case 13	Case 15	Case 16	Case 18	Diagnostic Accuracy	Score
M.D. 1	✗	✗	✗	✗	✗	✗	✓	✓	✗	2/9	2
M.D. 2	✗	✗	✗	✗	✗	✓	✗	✓	✗	2/9	2
M.D. 3	✗	✗	✗	✓	✓	✗	✗	✓	✓	4/9	4
M.D. 4	✗	✗	✗	✓	✓	✓	✗	✓	✗	4/9	4
M.D. 5	✗	✗	✗	✓	✗	✗	✗	✓	✗	2/9	2
M.D. 6	✗	✗	✗	✓	✗	✗	✗	✓	✗	2/9	2
M.D. 7	✗	✗	✗	✗	✗	✓	✗	✓	✗	2/9	2
M.D. 8	✗	✗	✗	✗	✗	✓	✗	✓	✗	2/9	2
M.D. 9	✗	✗	✗	✗	✗	✓	✗	✓	✓	3/9	3
M.D. 10	✗	✗	✗	✗	✗	✓	✗	✗	✓	2/9	2
Correct (n)	0/10	0/10	0/10	4/10	2/10	6/10	1/10	9/10	3/10	Median 2 [IQR 2–3]	

✓ = correct identification; ✗ = incorrect identification. No significant difference in diagnostic performance was observed among physicians (*p* = 0.73, Friedman test). IQR, interquartile range.

## Data Availability

The minimal dataset supporting the conclusions of this article is available as Supplementary Materials. The complete raw dataset is not publicly available due to patient privacy and confidentiality restrictions, in accordance with the ethical approval obtained from the Clinical Trial Review Board of Showa Medical University (Approval No. F2019C84). Further data are available from the corresponding author upon reasonable request.

## Supplementary Materials

The following supporting information can be downloaded at https://www.mdpi.com/article/10.3390/jcm15135071/s1, Table S1: Minimal anonymized dataset of the 25 patients with incidentally discovered malignancies, Table S2: Individual Diagnostic Performance of Ten Emergency Physicians Across Nine Missed Malignancy Cases.
